# Genetic Polymorphism rs6922269 in the MTHFD1L Gene Is Associated with Survival and Baseline Active Vitamin B12 Levels in Post-Acute Coronary Syndromes Patients

**DOI:** 10.1371/journal.pone.0089029

**Published:** 2014-03-11

**Authors:** Barry R. Palmer, Sandy Slow, Katrina L. Ellis, Anna P. Pilbrow, Lorraine Skelton, Chris M. Frampton, Suetonia C. Palmer, Richard W. Troughton, Tim G. Yandle, Rob N. Doughty, Gillian A. Whalley, Michael Lever, Peter M. George, Stephen T. Chambers, Chris Ellis, A. Mark Richards, Vicky A. Cameron

**Affiliations:** 1 Christchurch Heart Institute, Department of Medicine, University of Otago, Christchurch, New Zealand; 2 Genetics Otago, University of Otago, Christchurch, New Zealand; 3 Pathology Department, University of Otago, Christchurch, New Zealand; 4 Clinical Biochemistry Unit, Canterbury Health Laboratories, Christchurch, New Zealand; 5 Department of Medicine, Faculty of Medicine & Health Sciences, University of Auckland, Auckland, New Zealand; 6 Department of Medical Imaging, Unitec Institute of Technology, Auckland, New Zealand; 7 Institute of Food, Nutrition & Human Health, Massey University, Wellington, New Zealand; National Institute of Environmental Health Sciences, United States of America

## Abstract

**Background and Aims:**

The methylene-tetrahydrofolate dehydrogenase (NADP+ dependent) 1-like (*MTHFD1L*) gene is involved in mitochondrial tetrahydrofolate metabolism. Polymorphisms in *MTHFD1L*, including rs6922269, have been implicated in risk for coronary artery disease (CAD). We investigated the association between rs6922269 and known metabolic risk factors and survival in two independent cohorts of coronary heart disease patients.

**Methods and Results:**

DNA and plasma from 1940 patients with acute coronary syndromes were collected a median of 32 days after index hospital admission (Coronary Disease Cohort Study, CDCS). Samples from a validation cohort of 842 patients post-myocardial infarction (PMI) were taken 24–96 hours after hospitalization. DNA samples were genotyped for rs6922269, using a TaqMan assay. Homocysteine and active vitamin B12 were measured by immunoassay in baseline CDCS plasma samples, but not PMI plasma. All cause mortality was documented over follow-up of 4.1 (CDCS) and 8.8 (PMI) years, respectively. rs6922269 genotype frequencies were AA n = 135, 7.0%; GA n = 785, 40.5% and GG n = 1020, 52.5% in the CDCS and similar in the PMI cohort. CDCS patients with AA genotype for rs6922269 had lower levels of co-variate adjusted baseline plasma active vitamin B12 (p = 0.017) and poorer survival than patients with GG or GA genotype (mortality: AA 19.6%, GA 12.0%, GG 11.6%; p = 0.007). In multivariate analysis, rs6922269 genotype predicted survival, independent of established covariate predictors (p = 0.03). However the association between genotype and survival was not validated in the PMI cohort.

**Conclusion:**

*MTHFD1L* rs6922269 genotype is associated with active vitamin B12 levels at baseline and may be a marker of prognostic risk in patients with established coronary heart disease.

## Introduction

Acute coronary syndromes (ACS) are conditions that result from an acute reduction in perfusion of part of the coronary circulation, including unstable angina and non-ST- and ST-elevation myocardial infarction (MI). Genetic markers offer the promise of identifying individual patients at increased risks of early disease-onset, or worse prognosis, and who may benefit from additional screening and early intervention. Genetic polymorphisms have been associated with increased risk for ACS [1], [2], [3], but the number of reports investigating genetic association with clinical outcome following specific ACS events is comparatively few [4], [5], [6].

A genetic locus implicated in two independent genome-wide association studies (GWAS) of coronary artery disease (CAD) [7], [8] is the methylene-tetrahydrofolate dehydrogenase (NADP+ dependent) 1-like (*MTHFD1L*) gene. Single nucleotide polymorphisms (SNPs) from ∼20 kb of *MTHFD1L*, represented by the lead polymorphism, rs6922269, have been significantly associated with the development of CAD in several GWAS [7], [8]. The adjusted population attributable risk fraction for myocardial infarction (MI) from the German MI Family Study was reported as 11% (p = 0.009) for the rs6922269 A allele [8], and a recent meta-analysis confirmed its association with CHD risk [9].

Although rs6922269 has been associated with risk of developing CAD in several GWAS, genetic association studies of rs6922269 with clinical outcome in patients with established CAD are limited [10], [11]. We hypothesized that rs6922269 would be associated with mortality after hospital admission for ACS, and investigated its association with baseline characteristics, natriuretic peptides, other clinical risk factors and survival in a cohort of 1940 patients recruited after hospital admission for ACS and followed for over 4 years. We then sought validation of our findings in an independent cohort of 842 post-MI patients followed for 8.8 years. To investigate association with relevant metabolic risk factors we hypothesized that homocysteine and active vitamin B12 plasma levels would be perturbed in patients with the high-risk genotype of rs6922269, reflecting the role of MTHFD1L in folate metabolism. Availability of suitable plasma samples limited these folate pathway metabolite assays to a subset of the CDCS cohort.

## Methods

### Study population

#### Coronary Disease Cohort Study

The Coronary Disease Cohort Study (CDCS) recruited 1940 patients after admission to Christchurch or Auckland City Hospitals with a diagnosis of ACS, between July 2002 and January 2009. Inclusion criteria have been described previously [12]. Patients were excluded from the study if they had a severe co-morbidity that limited their life expectancy to <3 years (eg. end-stage renal failure, cancer). The aim of the study was to include a broad spectrum of age, both genders and significant sub-groups with established risk factors for CHD including those with hypertension and diabetes. Plasma was collected at a baseline clinic, at a median 32 days after the index admission for ACS. We collected demographic and clinical data at baseline including blood pressure, height, weight, ECG, echocardiography, family and personal medical history and medication. Plasma samples were assayed for BNP and NT-proBNP. Patients were followed for a median of 4.06 (range 0.12–7.92) years. Patients attended follow-up clinics 3–5 months and 12–14 months post-onset of ACS and participants completed questionnaires at two years and three years post-discharge. Standardized transthoracic echocardiography was performed using a GE Vivid 3 ultrasound system (GE Medical Systems, Auckland) at Christchurch Hospital and an ATL HDI 5000 (Philips Healthcare, Sydney) at Auckland City Hospital as described previously [5]. The New Zealand Multi-Region Ethics Committee approved the study. All participating patients provided written, informed consent.

#### Post-Myocardial Infarction Study

Patients (n = 842) were recruited into the Post-Myocardial Infarction (PMI) study between November 1994 and June 2001. The study assessed the prognostic utility of a range of circulating biomarkers after MI and has been described in detail elsewhere [13]. Acute MI (including ST elevation, STEMI, and non-ST elevation infarction, NSTEMI) was defined by the presence of typical cardiac ischemic symptoms, ischemic change in two or more contiguous EKG leads, and peak elevation of plasma creatine kinase >400 U/L. Patients were excluded if they were >80 years, had cardiogenic shock or did not survive the first 24 hours after admission. Although not an inclusion criterion, all patients were troponin T positive (0.1 µg/L). Median follow-up was 8.8 (0.1–13.0) years. The Canterbury Ethics Committee approved the study, and participating patients provided written informed consent.

### Clinical events

Clinical events were determined from recruitment questionnaires, planned follow-up clinic visits, consultation of patient notes, the National Health Information Service and hospital Patient Management System databases. Survival times were calculated from the date of index admission. The investigation conforms to the principles outlined in the Declaration of Helsinki and Title 45, U.S. Code of Federal Regulations, Part 46.

### Neurohormone and analyte measurements

Plasma samples were collected and stored in sealed tubes at −80°C. Circulating levels of natriuretic peptides were assayed as previously described [13]. NTproBNP plasma levels were stable for at least 18 months under these conditions. Plasma samples from each subject were analysed soon after the patient's 12-month clinic, so storage times from collection to analysis were similar in the two studies. Three sets of QC samples were measured in each assay and were overlapped with new QC samples for subsequent batches. No long-term drift was noted in the assay with QC samples. Sample storage is therefore unlikely to have influenced our results. Levels of creatine kinase (CK) and troponin T were measured using ELISA kits (Roche Diagnostics, Auckland). Homocysteine and active vitamin B12 were assayed by immunoassay on an automated Abbott ARCHITECT ci8200 Analyzer (Abbott Laboratories) by standard kit procedures in a laboratory accredited by International Accreditation New Zealand.

### DNA Extraction and rs6922269 polymorphism genotyping

Extraction of genomic DNA for genotyping was performed as described previously [5]. DNA samples were genotyped for the rs6922269 polymorphism in 10 µL reaction volumes using allele-specific TaqMan genotyping probes (Applied Biosystems, USA), including 1× Thermo qPCR ROX Mix (Thermo, UK) and 100 ng of genomic DNA in a Roche LC480 (Roche Diagnostics, Auckland).

### Statistical analysis

Univariate analyses to test for associations between rs6922269 genotype and demographic, neurohormonal and echocardiographic measurements were performed using χ^2^ and ANOVA tests. Skewed data were log-transformed before analysis and geometric means with 95% confidence intervals reported. The survival of genotype groups was compared using Kaplan-Meier analysis and the log-rank test. Independent associations between genotype and survival were tested using Cox proportional hazards multivariate analysis including the following established predictors; age [14], [15], gender [16], previous MI [15], antecedent hypertension [17], drug treatment [18], physical activity [19], and NT-proBNP levels [13]. We dichotomized plasma NT-proBNP levels above and below median values as previously described [20], to investigate any interaction between rs6922269 genotype and NT-proBNP levels. Ethnicity was self-declared and categorized as Maori/Pacific Islander, European, Other or Unknown. The study had power to detect a HR of >1.81 as statistically significant (two tailed p<0.05, 80% power) in the CDCS cohort. An additive genetic model was used unless stated otherwise. All analyses were performed using SPSS version 19 (SPSS Inc. Chicago, IL). Statistical significance was set at the 5% level (p<0.05).

## Results

### rs6922269 Genotype and CDCS cohort data

**Figure 1:**
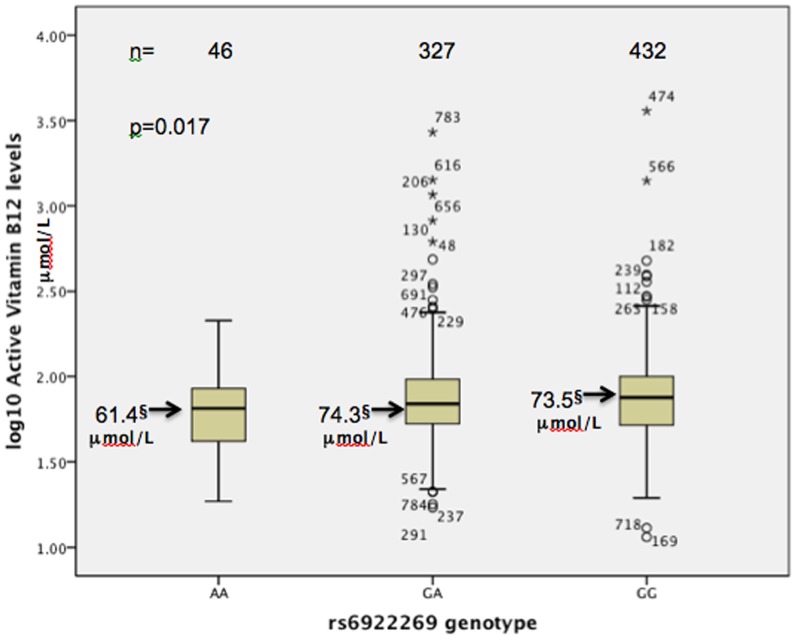
A boxplot comparison of log-transformed plasma active vitamin B12 levels at baseline post-admssion in rs6922269 genotype groups. The p-value compares data adjusted for age and hypertensive status. §Geometric means.

Genotypes were obtained for 1940 patients from the CDCS cohort for the *MTHFD1L* rs6922269 SNP. Baseline patient characteristics for each genotype group are shown in Table S1 in File S1. Genotype frequencies were AA, 7.0%; GA, 40.5% and GG, 52.5% (minor allele frequency = 0.272) and conformed to the Hardy-Weinberg equilibrium (p = 0.9998). Minor allele frequency (MAF) did not differ between ethnic groups in either cohort (p>0.134). Patients with AA genotype had markedly higher plasma creatinine at baseline (p = 0.008) and trended towards having lower LVEF (p = 0.086). Active vitamin B12 levels assayed from plasma samples taken at the patients' baseline clinic were lower in patients with AA genotype when adjusted for age and hypertensive status (Figure 1). Correction for multiple testing transformed these p-values to >0.05. No significant association with homocysteine levels at baseline was detected.

### rs6922269 Genotype and clinical outcome in the CDCS cohort

**Figure 2:**
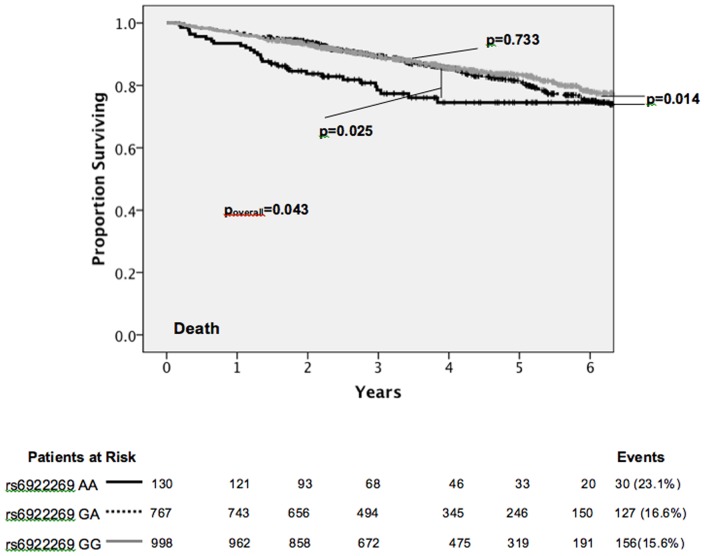
Kaplan-Meier survival analysis of CDCS cohort patients stratified by rs6922269 genotype.

**Figure 3:**
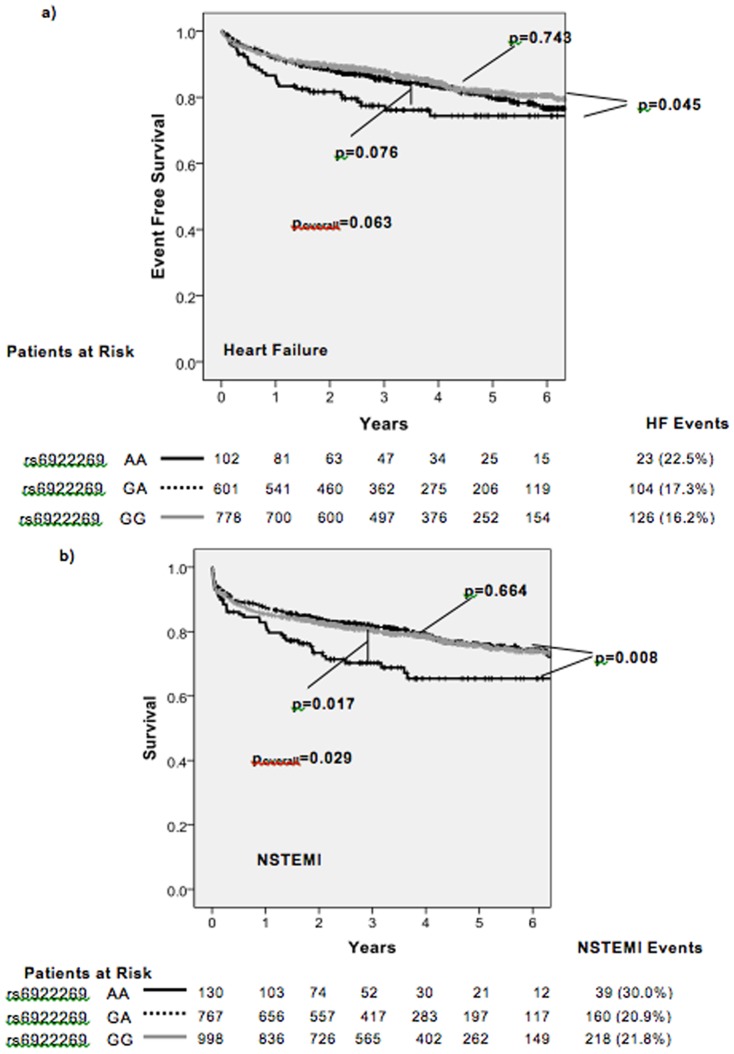
Kaplan-Meier analysis of event free survival to a) first HF admission, b) first NSTEMI admission stratified by rs6922269 genotype.

Survival in the patients with GG and GA genotypes was similar over the course of the follow-up period of a median of 4.06 (0.12–7.92) years, and was significantly greater than the AA patient group (mortality: AA 23.1%, GA 16.6%, GG 15.6%; p = 0.043) (Figure 2). An allelic test for association with survival was also significant (p = 0.035). rs6922269 genotype was significantly associated with first admission for NSTEMI (AA 28.1%, GA 21.5%, GG 22.4%; p = 0.029) and trended towards association with first admission for heart failure (AA 22.3%, GA 17.2%, GG 15.7%; p = 0.063) (Figure 3), but not admission for acute MI, STEMI, unstable angina or stroke (p>0.122).

**Figure 4:**
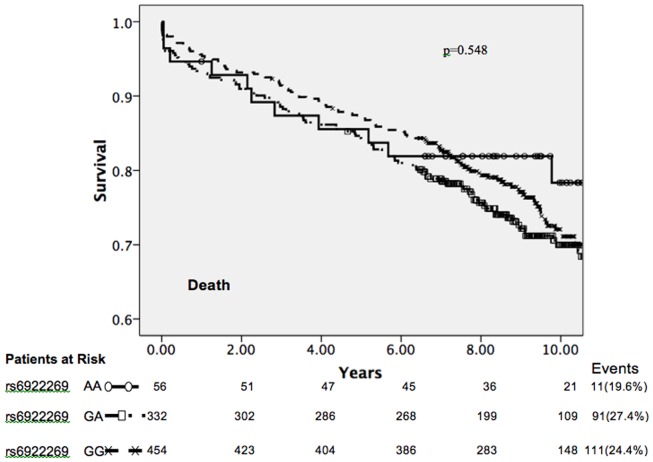
Kaplan-Meier survival analysis of CDCS cohort stratified by rs6922269 genotype a) in patients with above median BNP levels, b) in patients with below median BNP levels.

In multivariate analysis, rs6922269 genotype was an independent predictor of mortality in a Cox proportional hazards model including age, gender, ethnicity, antecedent hypertension, prior MI, physical activity score, statin and β-blocker treatment and plasma BNP levels (Table S2 in File S1). Genotype was a significant predictor of mortality (p_overall_ = 0.049; AA versus GG HR = 1.63, p = 0.025; GA versus GG HR = 1.22, p = 0.125; AA versus GA/GG HR = 1.23, p = 0.053). The Kaplan-Meier plots shown in Figure 4 illustrate that the majority of mortality occurred in the group of patients with above median NT-proBNP levels at baseline and genotype was significantly associated with mortality in this subgroup. This interaction was also significant (p = 0.001).

### rs6922269 Genotype and the PMI cohort

**Figure 5:**
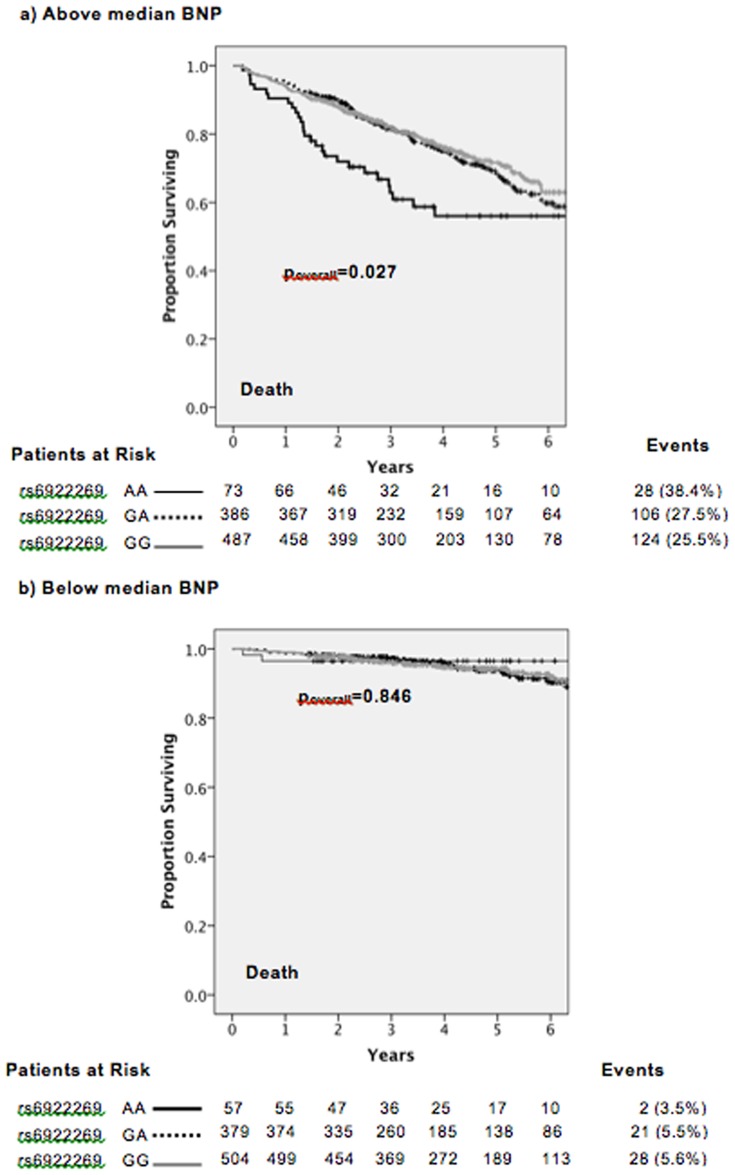
Kaplan-Meier survival analysis of PMI cohort patients stratified by rs6922269 genotype.

In order to validate the results from the CDCS cohort, 842 patients from the PMI cohort were genotyped for rs6922269. Genotype frequencies were AA 6.7%, GA 39.4% and GG 53.9% (MAF = 0.264). Patient characteristics for the PMI cohort stratified by genotype are shown in Table S3 in File S1. The prevalence of prior MI was greater in patients with an AA genotype (p = 0.025) and treatment at discharge with lipid lowering drugs trended towards being more common in this group also (p = 0.067). Survival over a median 8.8 (0.1–13.0) years follow-up was not significantly associated with rs6922269 genotype or alleles in the PMI cohort, either in a univariate (Figure 5) or multivariate analysis (Table S4 in File S1). Survival was not associated with genotype in either strata after dichotomization of the cohort by median NT-proBNP level (p>0.45), or presence or absence of a diagnosis of ST-elevation MI (p>0.56). However, if follow-up was truncated at 6 years and A-allele carriers grouped there was a trend towards an association between genotype and survival (n = 842, mortality: GG, 14.6% GA/AA 18.8%, p = 0.094). Suitable plasma samples were not available at the time of data generation, to allow assay for homocysteine and/or active vitamin B12 in the PMI study.

## Discussion

Mortality in patients with the AA genotype for rs6922269 was significantly greater than patients with GA or GG genotype in the CDCS cohort, limited to those patients with above median NT-proBNP levels at baseline, suggesting rs6922269 genotype may be useful in further risk stratification of these patients, already with a poor prognosis. Possibly only those with high NT-proBNP express the additional genetic risk of AA genotype, potentially mediated through vitamin B12 levels. Patients with an AA genotype had higher plasma creatinine levels and lower active vitamin B12 levels at baseline, indicating poorer kidney function and higher CVD risk. Inclusion of rs6922269 genotype in a Cox proportional hazards model including established predictors of mortality from similar cohorts, demonstrated genotype was an independent predictor of mortality.

The prognostic value of the rs6922269 genotype for mortality was not strongly replicated in the PMI cohort. While a hint of an allele-A dominant association with PMI cohort mortality prior to 6 years of follow-up was detected, this was much weaker than the association with mortality observed in the CDCS cohort. Differences between the cohorts may have obscured a genetic association, such as the much longer follow-up period, smaller size of the PMI cohort and a much more frequent diagnosis of STEMI in PMI patients (CDCS 9.6%; PMI 82.2%). However, subgroup analysis did not reveal an association with genotype in the PMI NSTEMI subgroup.

While the functional effect of rs6922269 is currently unknown, a possible mechanism through which *MTHFD1L* polymorphisms may affect CHD risk, and potentially clinical outcomes, is by influencing folate pathway metabolite levels. When adjusted for age and hypertensive status a significant association between active vitamin B12 levels and rs6922269 genotype is revealed. While correction for these particular covariates is not entirely intuitive, hypertension has been associated with reduced vitamin B12 levels [21]. Defects in the C1-tetrahydrofolate metabolic pathway may decrease the levels of vitamin B12. It is possible that the inconsistent findings associating rs6922269 with various heart disease phenotypes in this and other studies [7], [8], [10], [22] may be due in part to *MTHFD1L* gene-environment interactions, particularly dietary variation in folate content leading to variation in folate metabolite levels and therefore risks of disease progression. Folate status and drug treatment regimen have undergone substantial changes in New Zealand during the decade between the median dates of recruitment for each cohort [23], [24]. However it must be acknowledge that this interpretation of the data is circumstantial and as such should be viewed as hypothesis generating. Further studies of the utility of rs6922269 as a diagnostic and prognostic marker in CHD patients are needed, with particular emphasis on participants' dietary intake and nutritional status, especially folate pathway metabolites.

While a recent large meta-analysis of GWAS markers for dyslipidaemia, diabetes and CHD found rs6922269 was associated with CHD risk [9], investigation of the association of rs6922269 with survival in patients with established CAD has been limited. A recent GWAS of mortality after ACS identified rs6922269 as the only SNP of 95 investigated to achieve Bonferroni-corrected significant association with mortality in the test cohort (n = 811, p = 0.007). This finding was replicated in the African-American subgroup of a second cohort (p = 0.015), but not in two additional independent cohorts [11], resonant with our own results.

In conclusion *MTHFD1L* rs6922269 genotype proved to be an inconsistent marker of prognostic risk in two cohorts of patients with coronary heart disease, possibly because of gene-environment effects.

## Supporting Information

File S1 Table S1, Baseline characteristics for CDCS patients stratified by rs6922269 genotype. Table S2, Cox's proportional hazards regression model for mortality in the CDCS cohort (n = 1733, 281 deaths). Table S3, Baseline characteristics for PMI patients stratified by rs6922269 genotype. Table S4, Cox's proportional hazards regression model for mortality in the PMI cohort (n = 756, 192 deaths).
(DOCX)
