# Supplementary material for: Genetic Polymorphism rs6922269 in the MTHFD1L Gene Is Associated with Survival and Baseline Active Vitamin B12 Levels in Post-Acute Coronary Syndromes Patients
Source: PLoS One. 2014 Mar 11;9(3):e89029. doi: 10.1371/journal.pone.0089029 (PMC3949666; doi:10.1371/journal.pone.0089029)
Supplement: File S1 — Table S1, Baseline characteristics for CDCS patients stratified by rs6922269 genotype. Table S2, Cox's proportional hazards regression model for mortality in the CDCS cohort (n = 1733, 281 deaths). Table S3, Baseline characteristics for PMI patients stratified by rs6922269 genotype. Table S4, Cox's proportional hazards regression model for mortality in the PMI cohort (n = 756, 192 deaths). (DOCX) [file pone.0089029.s001.docx]

**Table S1. Baseline characteristics for CDCS patients stratified by rs6922269 genotype.**

rs6922269 genotype

|  | **n** | **AA** | **n** | **GA** | **n** | **GG** | **p-value** |
| --- | --- | --- | --- | --- | --- | --- | --- |
| Age (years)* | 130 | 68.5 ± 1.10 | 753 | 66.7 ± 0.43 | 985 | 66.5 ± 0.40 | 0.225 |
| Gender (F/M) | 135 | 41/94 | 785 | 235/550 | 1020 | 285/735 | 0.604 |
| Physical Activity (scored 1-4) *† | 130 | 2.98±0.11 | 747 | 3.03±0.04 | 982 | 2.92±0.04 | 0.145 |
| BMI (kg/m^2^) * | 125 | 26.8±0.39 | 744 | 27.4±0.17 | 975 | 27.6±0.17 | 0.179 |
| LVEF* | 126 | 55.8±1.05 | 718 | 57.8±0.43 | 927 | 56.7±0.42 | 0.086 |
| *History* |  |  |  |  |  |  |  |
| Previous myocardial infarction* | 129 | 47(36.4%) | 752 | 209(27.8%) | 982 | 302(30.8%) | 0.103 |
| hypertension * | 130 | 75(57.76%) | 747 | 408(54.6%) | 985 | 497(50.5%) | 0.112 |
| Type II Diabetes* | 131 | 25(19.1%) | 751 | 118(15.7%) | 987 | 168(17.0%) | 0.567 |
| Renal Disease* | 129 | 15(11.6%) | 761 | 75(9.9%) | 995 | 95(9.5%) | 0.756 |
| *Laboratory Data* |  |  |  |  |  |  |  |
| Total Cholesterol‡ (mmol/l) | 58 | 4.78(4.50-5.07) | 403 | 4.81(4.67-4.95) | 506 | 4.80(4.69-4.91) | 0.985 |
| Plasma Creatinine‡ (mmol/l) | 95 | 0.131(0.097-0.177) | 601 | 0.100(0.096-0.105) | 756 | 0.108(0.102-0.115) | 0.008 |
| Peak Creatine kinase‡ (units/l) | 89 | 776(144-296) | 574 | 246(219-277) | 738 | 219(198-244) | 0.289 |
| NT-proBNP (pmol/l)‡ | 133 | 88.6 (73.4-107) | 767 | 80.0(71.9-83.3) | 997 | 74.5(69.6-79.8) | 0.204 |
| BNP (pmol/l)‡ | 132 | 19.0(16.4-22.1) | 766 | 16.9(15.8-18.0) | 996 | 17.1(16.1-18.1) | 0.371 |
| Homocysteine (mmol/l)* | 48 | 14.0±0.77 | 331 | 14.2±0.32 | 442 | 13.6±0.25 | 0.404 |
| Active vitamin B12 (mmol/l)* | 48 | 64.1±4.67 | 337 | 74.5±1.76 | 451 | 75.1±1.52 | 0.081 |
| *Discharge Medications* |  |  |  |  |  |  |  |
| ACE inhibitor * | 131 | 70(53.4%) | 769 | 434(56.4%) | 1003 | 568(56.6%) | 0.784 |
| β-blocker * | 131 | 114(87.0%) | 769 | 662 (86.1%) | 1003 | 861(85.8%) | 0.933 |
| Diuretic* | 131 | 38(29.0%) | 769 | 199(25.9%) | 1003 | 265(26.4%) | 0.753 |
| Statin* | 131 | 110(84.0%) | 769 | 676(87.9%) | 1003 | 862(85.9%) | 0.319 |

*Data are means (SEM) or occurrence (percentage); †Score of 1 = sedentary, 2 = <30 mins activity on >2 days/week, 3 = ≥30 mins on 2 days/week, 4 = ≥30 mins on ≥3 days/week ; ‡Geometric mean (95% confidence interval).

**Table S2. Cox’s proportional hazards regression model for mortality in the CDCS cohort (n=1733, 281 deaths).**

|  | Coefficient | SE | Wald | df | Significance | Hazard Ratio | 95% CI for HR | |
| --- | --- | --- | --- | --- | --- | --- | --- | --- |
|  |  |  |  |  |  |  | Lower | Upper |
| Age at index admission | 0.05 | 0.01 | 61.8 | 1 | <0.001 | 1.06 | 1.04 | 1.07 |
| Male Gender | 0.12 | 0.07 | 2.86 | 1 | 0.09 | 1.12 | 0.98 | 1.28 |
| Log_10_ BNP* | 1.53 | 0.20 | 59.1 | 1 | <0.001 | 4.60 | 3.12 | 6.80 |
| LVEF | -0.01 | 0.01 | 3.94 | 1 | 0.047 | 0.99 | 0.98 | 1.00 |
| β-blocker treatment at discharge | -0.51 | 0.16 | 10.2 | 1 | 0.001 | 0.60 | 0.44 | 0.82 |
| Physical Activity (scale 1-4)† | -0.29 | 0.05 | 34.4 | 1 | <0.001 | 0.75 | 0.68 | 0.82 |
| Previous Myocardial Infarction | 0.44 | 0.13 | 12.4 | 1 | <0.001 | 1.56 | 1.22 | 2.00 |
| Antecedent Hypertension | 0.44 | 0.13 | 11.3 | 1 | 0.001 | 1.55 | 1.20 | 2.00 |
| Ethnicity |  |  | 8.03 | 3 | 0.045 |  |  |  |
| European versus Maori/Pacific Islanders | 0.68 | 0.25 | 7.44 | 1 | 0.006 | 1.98 | 1.21 | 3.23 |
| European versus Other‡ | 0.41 | 0.46 | 0.80 | 1 | 0.371 | 1.51 | 0.61 | 3.74 |
| European versus Unknown | 0.23 | 0.51 | 0.20 | 1 | 0.654 | 1.26 | 0.46 | 3.40 |
| rs6922269 genotype |  |  | 6.03 | 2 | 0.049 |  |  |  |
| AA versus GG | 0.49 | 0.22 | 5.01 | 1 | 0.025 | 1.63 | 1.06 | 2.51 |
| AA versus GA | 0.20 | 0.13 | 2.35 | 1 | 0.125 | 1.22 | 0.95 | 1.57 |

*Hazard Ratio represents the change in risk for every 10-fold increase in BNP level. †Score of 1=sedentary, 2=<30 minutes activity on >2 days/week, 3=≥30 minutes on 2 days/week, 4= ≥30 minutes on ≥3 days/week. ‡Includes Asian, South Asian, Middle Eastern and African.

**Table S3. Baseline characteristics for PMI patients stratified by rs6922269 genotype.**

rs6922269 genotype

|  | **n** | **AA** | **n** | **GA** | **n** | **GG** | **p-value** |
| --- | --- | --- | --- | --- | --- | --- | --- |
| Age (years)* | 56 | 62.2±1.49 | 332 | 63.2 ± 0.55 | 454 | 61.6 ± 0.50 | 0.103 |
| Gender (F/M) | 56 | 16/40 | 332 | 68/264 | 454 | 98/356 | 0.396 |
| BMI (kg/m^2^)* | 54 | 26.6±0.59 | 317 | 26.2±0.20 | 433 | 26.8±0.19 | 0.095 |
| LVEF* | 50 | 46.9±1.52 | 304 | 47.9±0.70 | 415 | 47.4±0.60 | 0.793 |
| *History* |  |  |  |  |  |  |  |
| Previous myocardial infarction* | 56 | 15(26.8%) | 332 | 44(13.3%) | 454 | 81(17.8%) | 0.025 |
| hypertension * | 56 | 25(44.6%) | 332 | 119(35.8%) | 454 | 174(38.3%) | 0.425 |
| Type II Diabetes* | 56 | 10(17.9%) | 344 | 49(14.8%) | 454 | 49(10.8%) | 0.132 |
| Renal Disease* | 46 | 3(6.5%) | 278 | 13(4.7%) | 373 | 17(4.6%) | 0.838 |
| *Laboratory Data* |  |  |  |  |  |  |  |
| Total Cholesterol* (mmol/l) | 52 | 5.91±0.18 | 323 | 5.88±0.07 | 427 | 5.92± 0.06 | 0.900 |
| Plasma Creatinine* (mmol/l) | 56 | 0.086± 0.003 | 332 | 0.089± 0.002 | 454 | 0.087± 0.001 | 0.659 |
| Peak Creatine kinase† (units/l) | 56 | 1340(1110-1610) | 332 | 1615 (1490-1750) | 467 | 1600 (1500-1710) | 0.182 |
| NT-proBNP† (pmol/l) | 54 | 101 (81.0-126) | 324 | 113(104-123) | 448 | 113(105-121) | 0.598 |
| *Discharge Medications* |  |  |  |  |  |  |  |
| ACE inhibitor * | 56 | 25(44.6%) | 332 | 166(50.0%) | 454 | 219(48.2%) | 0.729 |
| β-blocker * | 56 | 46(82.1%) | 332 | 279(84.0%) | 454 | 382(84.1%) | 0.928 |
| Diuretic* | 56 | 14(25.0%) | 332 | 63(19.0%) | 454 | 93(20.5%) | 0.568 |
| Lipid lowering* | 56 | 34(60.7%) | 332 | 146(44.0%) | 454 | 211(46.5%) | 0.067 |

*Data are means (SEM) or occurrence (percentage); †Geometric mean (95% confidence interval).

**Table S4. Cox’s proportional hazards regression model for mortality in the PMI cohort (n=756, 192 deaths).**

|  | Coefficient | SE | Wald | df | Significance | Hazard Ratio | 95% CI for HR | |
| --- | --- | --- | --- | --- | --- | --- | --- | --- |
|  |  |  |  |  |  |  | Lower | Upper |
| Age at index admission | 0.05 | 0.01 | 30.9 | 1 | <0.001 | 1.06 | 1.04 | 1.08 |
| Male Gender | 0.08 | 0.18 | 0.16 | 1 | 0.687 | 1.07 | 0.76 | 1.51 |
| Log_10_ BNP* | 0.50 | 0.13 | 14.9 | 1 | <0.001 | 1.64 | 1.28 | 2.12 |
| LVEF | -0.02 | 0.01 | 9.47 | 1 | 0.002 | 0.98 | 0.96 | 0.99 |
| β-blocker treatment at discharge | 0.33 | 0.18 | 3.43 | 1 | 0.064 | 1.40 | 0.98 | 1.98 |
| Previous Myocardial Infarction | 0.65 | 0.17 | 15.0 | 1 | <0.001 | 1.90 | 1.37 | 2.65 |
| Antecedent Hypertension | 0.16 | 0.15 | 0.01 | 1 | 0.917 | 1.02 | 0.76 | 1.36 |
| Ethnicity |  |  | 5.04 | 3 | 0.169 |  |  |  |
| European versus Maori/Pacific Islanders | 0.69 | 0.43 | 2.54 | 1 | 0.11 | 1.99 | 0.85 | 4.65 |
| European versus Other‡ | -0.60 | 0.39 | 2.39 | 1 | 0.12 | 0.55 | 0.26 | 1.17 |
| European versus Unknown | -0.02 | 0.25 | 0.01 | 1 | 0.94 | 0.98 | 0.60 | 1.60 |
| rs6922269 genotype |  |  | 0.82 | 2 | 0.66 |  |  |  |
| AA versus GG | -0.13 | 0.34 | 0.15 | 1 | 0.70 | 0.88 | 0.46 | 1.70 |
| AA versus GA | 0.11 | 0.15 | 0.53 | 1 | 0.47 | 1.12 | 0.83 | 1.51 |

*Hazard Ratio represents the change in risk for every 10-fold increase in BNP level. ‡Includes Asian, South Asian, Middle Eastern and African.
